# Matrix-Tolerant Quantification of THC and THCA in Complex Cannabis Products Using In-Sample Calibration with Multiple Isotopologue Reaction Monitoring

**DOI:** 10.1021/jasms.5c00439

**Published:** 2026-01-26

**Authors:** Yanfang Li, Mengliang Zhang

**Affiliations:** Department of Chemistry and Biochemistry, 1354 Ohio University, Athens, Ohio 45701, United States

**Keywords:** cannabis, in-sample calibration curve (ISCC), multiple isotopologue reaction monitoring (MIRM), Δ^9^-tetrahydrocannabinol (Δ^9^-THC), Δ^9^-tetrahydrocannabinolic acid (THCA)

## Abstract

Accurate quantification of Δ^9^-tetrahydrocannabinol (THC) and Δ^9^-tetrahydrocannabinolic acid (THCA) across diverse cannabis- and hemp-derived products remains challenging due to severe matrix effects, wide concentration variability, and the need for matrix-matched calibration in traditional LC–MS workflows. Here, we develop an in-sample calibration curve (ISCC) method based on multiple isotopologue reaction monitoring (MIRM) from stable-isotope-labeled (SIL) analytes to enable robust quantification of THC and THCA without external calibration curves. The approach leverages the theoretical relative isotopic abundances of SIL calibrators to generate multiple internal calibration points within each injection. By incorporating two SIL calibrators for THC (THC-D_3_ and THC-D_9_), applying a response-correction factor to harmonize labeled and unlabeled analytes, and utilizing native-analyte isotopologue transitions at high abundance, the method achieves a >600-fold dynamic range. The ISCC method demonstrated excellent linearity (*R* ^2^ > 0.999), precision (<10% RSD), and accuracy (±10%) in commercial CBD oils, gummies, creams, waxes, dietary supplements, and plant materials. Comparison with external calibration showed strong agreement across all matrices. Collectively, this work develops the ISCC–MIRM framework for heterogeneous consumer and forensic samples and establishes a practical, matrix-tolerant calibration strategy for routine cannabinoid analysis.

## Graphical abstract

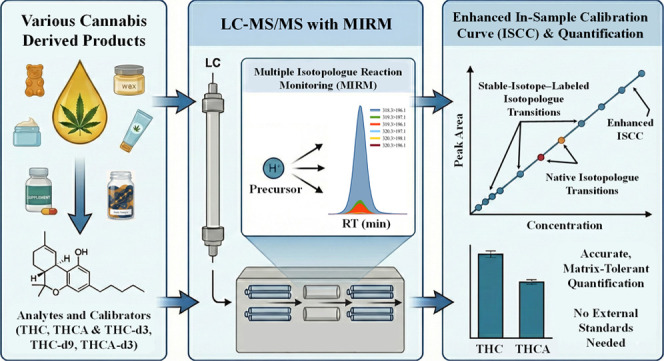

## Introduction

The increasing availability of cannabis and hemp-derived consumer products has created a pressing need for accurate and reliable quantification of Δ^9^-tetrahydrocannabinol (THC), Δ^9^-tetrahydrocannabinolic acid (THCA), and related cannabinoids across a wide range of product matrices.^1–3^ Regulatory frameworks in the United States, including the 2018 Agriculture Improvement Act, legally define hemp as containing less than 0.3% total THC (the sum of Δ^9^-THC and Δ^9^-THCA), making quantitative chemical analysis essential for distinguishing compliant hemp products from controlled cannabis materials.^4^ As a result, forensic laboratories are increasingly required to perform high-throughput cannabinoid testing across chemically diverse, often highly complex matrices such as plant material, edibles, oils, creams, waxes, and dietary supplements. These matrices frequently introduce substantial matrix effects, including ion suppression and nonlinear detector response, posing significant challenges for conventional LC-MS-based quantification workflows.^5^

Triple-quadrupole LC–MS/MS is a well-established method for cannabinoid quantification. However, this approach generally relies on multipoint external calibration curves and matrix-matched standards to compensate for variable matrix effects.^6,7^ Although stable isotope-labeled internal standards mitigate many sources of variability, quantitative cannabinoid analysis in heterogeneous consumer products often still relies on matrix-matched external calibration. This is because matrix effects can vary substantially across product categories (e.g., plant, gummies, oils, waxes, creams), and suppression/enhancement can be analyte- and matrix-specific even when isotope-labeled standards are used. Consequently, laboratories frequently maintain multiple matrix-matched calibration sets and dilution schemes to cover diverse sample types and wide concentration ranges, which increases analytical burden and can still leave residual bias when a calibration matrix does not sufficiently represent the unknown sample. Preparing multipoint calibration sets is also labor-intensive and increases analytical cost. Moreover, THC and THCA concentrations can vary by several orders of magnitude across commercial samples, often necessitating repeated dilutions, additional calibration levels, or separate analytical runs, all of which reduce efficiency. Recent proficiency testing through the Cannabis Laboratory Quality Assurance Program (CannaQAP) conducted by NIST highlighted substantial interlaboratory variability and bias, particularly at lower cannabinoid concentrations.^8^ Increasing matrix diversity in cannabis-derived products further complicates accurate quantitation and the need for separate validations across product categories imposes an additional burden on forensic and regulatory laboratories.^9,10^

To address these challenges, we developed an in-sample calibration curve (ISCC) strategy based on multiple isotopologue reaction monitoring (MIRM) transitions from stable-isotope-labeled (SIL) analytes for quantifying THC and THCA in cannabis-derived products. The ISCC–MIRM approach was originally introduced by Gu et al. for quantitative analysis of small-molecule drugs, peptides, and proteins in biological matrices.^11,12^ Their work demonstrated that multiple isotopologue transitions from a single stable-isotope-labeled (SIL) calibrator can generate an internal calibration curve without external standards. However, the method had not previously been adapted to heterogeneous consumer or forensic matrices, nor applied to analytes with the extreme dynamic concentration ranges characteristic of THC and THCA. In the present study, we extend the ISCC framework in several important ways: (i) by incorporating two SIL calibrators (THC-D_3_ and THC-D_9_) to accommodate >600-fold concentration variability encountered in cannabis-derived products; (ii) by introducing a correction-factor procedure to harmonize response differences between labeled and unlabeled analytes arising from isotopic substitution, purity variation, and molar-to-mass conversion; and (iii) by utilizing isotopologue transitions of native THC and THCA as quantitative channels when their primary monoisotopic transitions saturate at high abundance. These methodological advances enable the ISCC method to be applied reliably across oils, edibles, waxes, creams, plant materials, and dietary supplements, with matrices far more complex and heterogeneous than those evaluated in earlier ISCC studies.

In this work, we evaluate the ISCC method using THC-D_3_, THC-D_9_, and THCA-D_3_ as internal calibrators and apply the approach to a range of commercial CBD products and cannabis plant materials. Linearity, precision, accuracy, and matrix effects are systematically assessed across multiple product categories, and ISCC performance is compared with that of a conventional external calibration method. The results demonstrate that ISCC provides accurate, matrix-tolerant quantification of THC and THCA with minimal sample-preparation burden and without reliance on matrix-matched external standards. Collectively, this work establishes ISCC as a practical, efficient, and broadly applicable alternative to traditional calibration strategies for routine analysis of cannabinoids in complex forensic and commercial matrices.

## Materials and Methods

### Chemicals

LC–MS grade acetonitrile (ACN), methanol, formic acid, and HPLC grade water were obtained from Fisher Scientific, Inc. (Fair Lawn, NJ, USA). Δ^9^-Tetrahydrocannabinol (THC) and cannabidiol (CBD) standard solutions at 1 mg/mL were purchased from Cerilliant Corporation (Round Rock, TX). Δ^9^-Tetrahydrocannabinolic acid (THCA), Δ^9^-THC-D_3_, Δ^9^-THC-D_9_, and Δ^9^-THCA-D_3_ were purchased from Cayman Chemical (Ann Arbor, MI, USA). QuEChERS extract pouch was purchased from Agilent Technologies (Palo Alto, CA, USA).

### Sample Materials

Cherry hemp, CBD gummy, CBD capsules (oil), CBD lip and body balm (wax), CBD cream, and a CBD dietary supplement (oil/cream-like texture) were obtained from a local CBD retailer in Murfreesboro, TN. Two legacy plant materials (originally labeled as “marijuana” in 2009) were obtained from the Analytical/Forensic Chemistry Laboratory at Ohio University; both samples were verified to contain <0.3% total THC.

### Preparation of Standard Mixtures

Four mixtures were prepared for ISCC construction and evaluation of matrix effects.

Mixture 1 (SIL calibrators): THC-D_9_, THC-D_3_, and THCA-D_3_ were diluted in 0.1% formic acid in methanol to yield final concentrations of 5.0, 1.5, and 1.5 μg/mL, respectively, after a 10-fold dilution into sample solutions. Mixture 2–4 (native analytes): THC, THCA, and CBD were diluted at multiple levels to generate working solutions with final concentrations ranging from 0.5 to 7.5 μg/mL. Specifically, Mixture 2 has the final concentrations of 2.5 μg/mL THC, 2.5 μg/mL CBD, and 0.5 μg/mL THCA; Mixture 3 has the final concentrations of 7.5 μg/mL THC and 1.5 μg/mL THCA; and Mixture 4 has the final concentrations of 1.5 μg/mL THC and 0.5 μg/mL THCA. These mixtures were combined with Mixture 1 at defined ratios to prepare the solutions used for ISCC calibration and matrix-effect studies.

### ISCC Working Solutions

ISCC working solutions were prepared by combining Mixture 1 with either Mixture 2 or Mixture 3 at a 90:10 (v/v) ratio. These produced solutions containing constant levels of SIL calibrators (THC-D_3_, THC-D_9_, THCA-D_3_) and variable concentrations of the native analytes (THC, THCA, CBD).

### Sample Preparation for Matrix Effects

Different sample preparation procedures were used for plant, gummy, and oily/wax/cream matrices, based on the existing literature, given their differing physical and chemical characteristics. *Plant material:* 50 mg was extracted with 10 mL of methanol by vortexing for 1 min and sonication for 20 min. After settling, 1 mL of supernatant was filtered through a 0.22 μm nylon syringe filter.^13^ *Gummy:* Finely chopped gummy (50 mg) was extracted with 10 mL water, followed by 10 mL ACN, vortexed for 1 min, sonicated for 20 min, then added to a QuEChERS extract pouch packet (containing 4 g magnesium sulfate and 1 g sodium chloride), and vortexed for 1 min. After standing for 10 min, the ACN layer was collected and filtered.^1^ *Oil, wax, cream, dietary supplement:* An additional defatting step was necessary for fat-rich samples to reduce the matrix interference, and the protocol involving hexane defattening was adopted from the QuEChERS manufacturer’s procedure.^14,15^ In short, 50 mg of sample was weighed into a 50 mL centrifuge tube with 10 mL of hexane and vortexed for 1 min. Then 10 mL of water was added, vortexed for 1 min, and sonicated for 20 min. After sonication, 10 mL acetonitrile was added, vortexed for 1 min, then a QuEChERS pouch was added, followed by a final 1 min vortex. After standing for 10 min, four layers formed (top: hexane; second: acetonitrile; third: aqueous/suspension; bottom: precipitated salts). The top hexane layer was carefully removed, and a 1 mL aliquot of the acetonitrile layer was collected and filtered through a 0.22 μm nylon syringe filter. All the samples were extracted in triplicate. For matrix-effect evaluation, extracts (50 μL) were combined with 50 μL of Mixture 4 and 900 μL of Mixture 1. Control samples containing no added THC or THCA were prepared to assess endogenous levels in commercial products.

### Sample Preparation for Precision and Accuracy Evaluation

CBD oil and gummy samples were spiked at three levels (0.03%, 0.10%, and 0.30%) with THC, THCA, and their SIL calibrators prior to extraction (Table S1). Following extraction, each sample was diluted (1:10) with methanol and analyzed using the ISCC workflow. All samples were prepared in triplicate.

### Sample Preparation for Comparison of ISCC and External Calibration

Commercial CBD samples and cannabis plant materials were fortified at multiple THC and THCA levels (0.003–1.0% and 0.003–0.5%, respectively) as listed in Table S2. Extracts were diluted with Mixture 1 prior to instrumental analysis. For external calibration, mixed THC/THCA standards (0.005–5 μg/mL) were prepared in methanol.

### LC–QQQ–MS Method

The LC–QQQ–MS was conducted using a Shimadzu LCMS-8050 system, including a DGU-20A degassing unit, LC-20AD, SIL-20AC autosampler, CBM-20A communication module, CTO-20A column oven, and a triple quadrupole mass spectrometer (Shimadzu Corporation, Columbia, MD). The MS was equipped with a dual unique ionization source (DUIS), operating in positive-ion mode for THC, THC-D_3_, and THC-D_9_, and in negative-ion mode for THCA and THCA-D_3_. The conditions for MS analysis were as follows: interface voltage, DUIS­(+): 4.0 kV; DUIS(−): −3.0 kV; desolvation line temperature, 250 °C; heat block temperature, 400 °C; interface temperature, 300 °C; nebulizing gas flow, 2 L/min; drying gas flow, 10 L/min; and heating gas flow, 10 L/min. The LC gradient was adopted from our previous work and provided sufficient separation for 11 cannabinoids.^16^ In short, a UPLC BEH shield RP18 column (50 × 2.1 mm inner diameter, 1.7 μm) (Waters, Milford, MA) was used with an UltraLine UHPLC In-Line Filter (RESTEK, Bellefonte, PA). The flow rate was 0.3 mL/min with an injection volume of 1 μL. Mobile phase A was 0.2% formic acid in water, and mobile phase B was acetonitrile. The linear gradient started at 50% B (v/v) and held for 2 min, increased to 85% B over 5 min, held at 85% B for 10.5 min, then decreased to 50% B at 10.6 min, and held at 50% B for 12 min. MIRM transitions’ parameters (i.e., Dwell time, *Q*1/*Q*3 voltages, and collision energy (CE)) for all native analytes and SIL calibrators are summarized in Table S3, and their theoretical isotopologue distributions are listed in Table S4. Theoretical isotopic distributions of the fragment ion and neutral loss were obtained through an online isotopic distribution calculator (https://www.sisweb.com/mstools/isotope.htm). The most abundant transitions (i.e., the monoisotopic transitions) were used for matrix-effect evaluation, while all isotopologue transitions were used for ISCC construction.

## Results and Discussion

### Theory and Construction of the In-Sample Calibration Curve (ISCC)

The ISCC quantification strategy is based on the predictable isotopic distributions of stable-isotope-labeled (SIL) analytes and the ability to monitor these distributions through multiple isotopologue reaction monitoring (MIRM) transitions. In this work, Δ^9^-THC-D_3_, Δ^9^-THC-D_9_, and Δ^9^-THCA-D_3_ were selected as SIL internal calibrators. For each SIL compound, the most abundant MIRM transition (precursor → fragment ion) was used as the primary reference channel (e.g., *m*/*z* 318.3 → 196.1 for THC-D_3_), and adjacent isotopologue transitions arising from natural carbon/hydrogen isotopic variants were simultaneously monitored.

Figure 1 illustrates the conceptual framework using Δ^9^-THC-D_3_ as an example. THC-D_3_ produces a monoisotopic precursor ion at *m*/*z* 318.3 ([M + H]^+^), which fragments predominantly to a product ion at *m*/*z* 196.1 through loss of C_9_H_14_ (122.1 Da). Because both the precursor ion and fragment ion contain several carbon and hydrogen atoms, natural isotopic substitutions (e.g., ^13^C, ^2^H) generate a series of isotopologues. These isotopologues give rise to a set of additional MIRM channels offset by +1 or +2 Da in either the precursor or product ion mass. For THC-D_3_, six isotopologue transitions were selected (*m*/*z* 318.3 → 196.1, 319.3 → 196.1, 319.3 → 197.1, 320.3 → 196.1, 320.3 → 197.1, 320.3 → 198.1). The theoretical isotopic abundance of each transition can be calculated directly from the molecular formulas of the neutral-loss fragment and product ion (Figure 1A). Specifically, the probability of observing a given precursor → product isotopologue pair is equal to the product of (i) the relative isotopic abundance of the neutral-loss fragment and (ii) the relative isotopic abundance of the product-ion isotopologue (Figure 1B). The calculated abundances, obtained from an isotopic distribution calculator, are summarized in Table S3. For example, the *m*/*z* 319.3 → 197.1 channel corresponds to a neutral-loss abundance of 9.90% and a fragment-ion abundance of 13.22%, yielding a predicted relative transition abundance of 1.31%. To verify that the calculated isotopic distributions accurately reflect the actual mass spectrometric behavior of the SIL analyte, the theoretical isotopic abundances of each MIRM transition were compared with the experimentally measured isotopic abundances obtained from THC-D_3_ (Figure 1C). The agreement (*R* ^2^ = 1.000) confirms that the predicted isotopic pattern is faithfully reproduced in the experimental data. This validation demonstrates that theoretical isotopic abundances can be used reliably in subsequent steps to calculate the theoretical concentration equivalents required for ISCC construction. For THC-D_3_, these channels span a ∼250-fold range in theoretical abundance while maintaining excellent linearity (*R* ^2^ = 1.000). These validated theoretical relative abundances are then converted into “theoretical concentration equivalents” by assigning each isotopologue transition a concentration proportional to its relative abundance with respect to the primary reference channel. These concentration equivalents serve as the *x*-values for constructing the ISCC in subsequent calibration (Figure 2).

**Figure 1:**
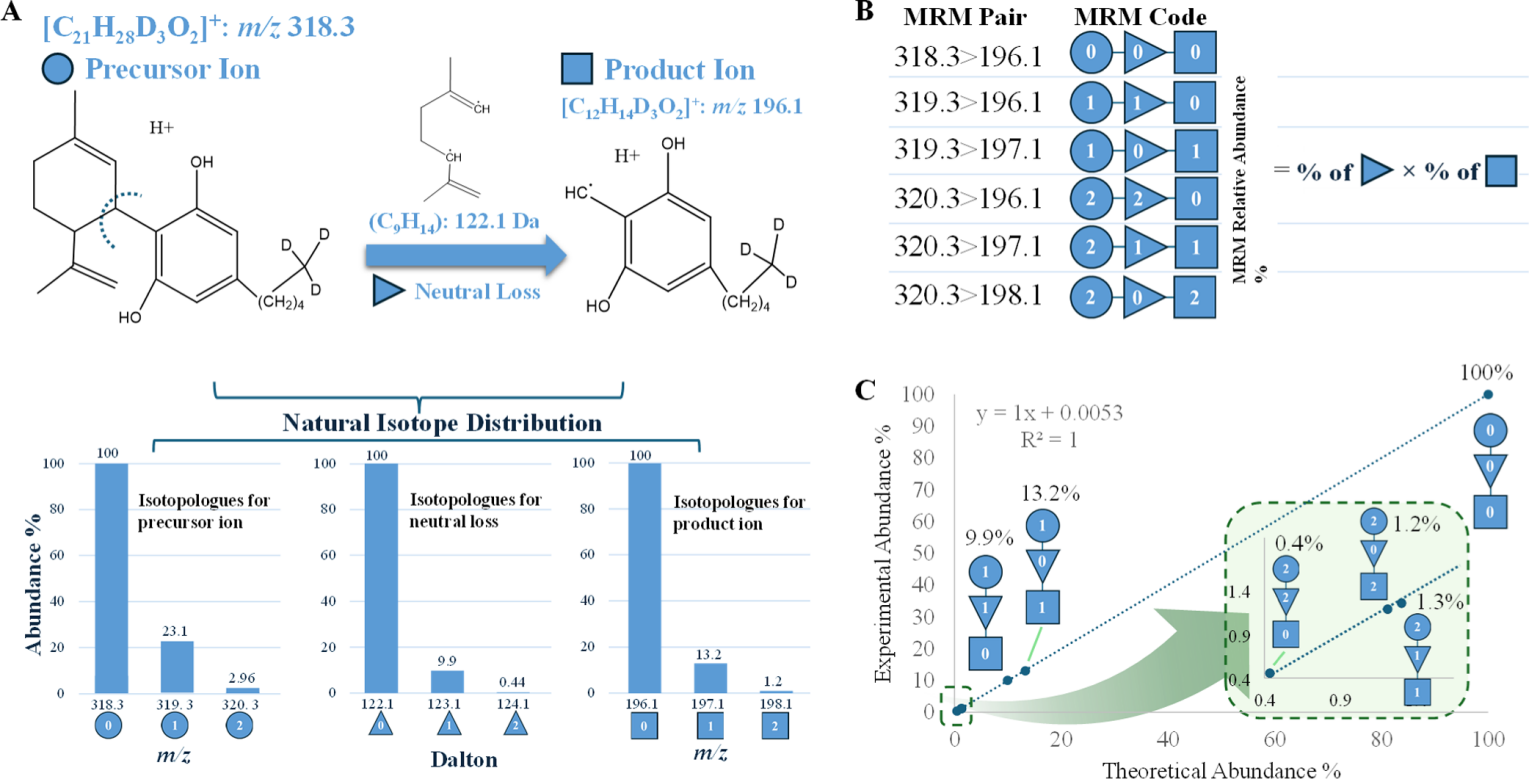
Scheme illustrating the theoretical basis of the ISCC approach using THC-D_3_ as an example. (A) Protonated precursor ion of THC-D_3_ (C_21_H_27_D_3_O_2_; [M + H]^+^ at *m*/*z* 318.3), its characteristic neutral loss (C_9_H_14_; 122.1 Da), and the resulting product ion ([C_12_H_14_D_3_O_2_]^+^ at *m*/*z* 196.1). The lower panel shows the theoretical *relative isotopic distributions* of the precursor, neutral-loss fragment, and product ion. (B) Calculation of the theoretical *relative isotopic abundance* for each MIRM isotopologue transition. For a given precursor → product pair, the theoretical abundance equals: (relative isotopic abundance of the neutral-loss fragment) × (relative isotopic abundance of the product-ion isotopologue). (C) Comparison of theoretical and experimentally measured *relative isotopic abundances* for the six MIRM transitions of THC-D_3_.

**Figure 2:**
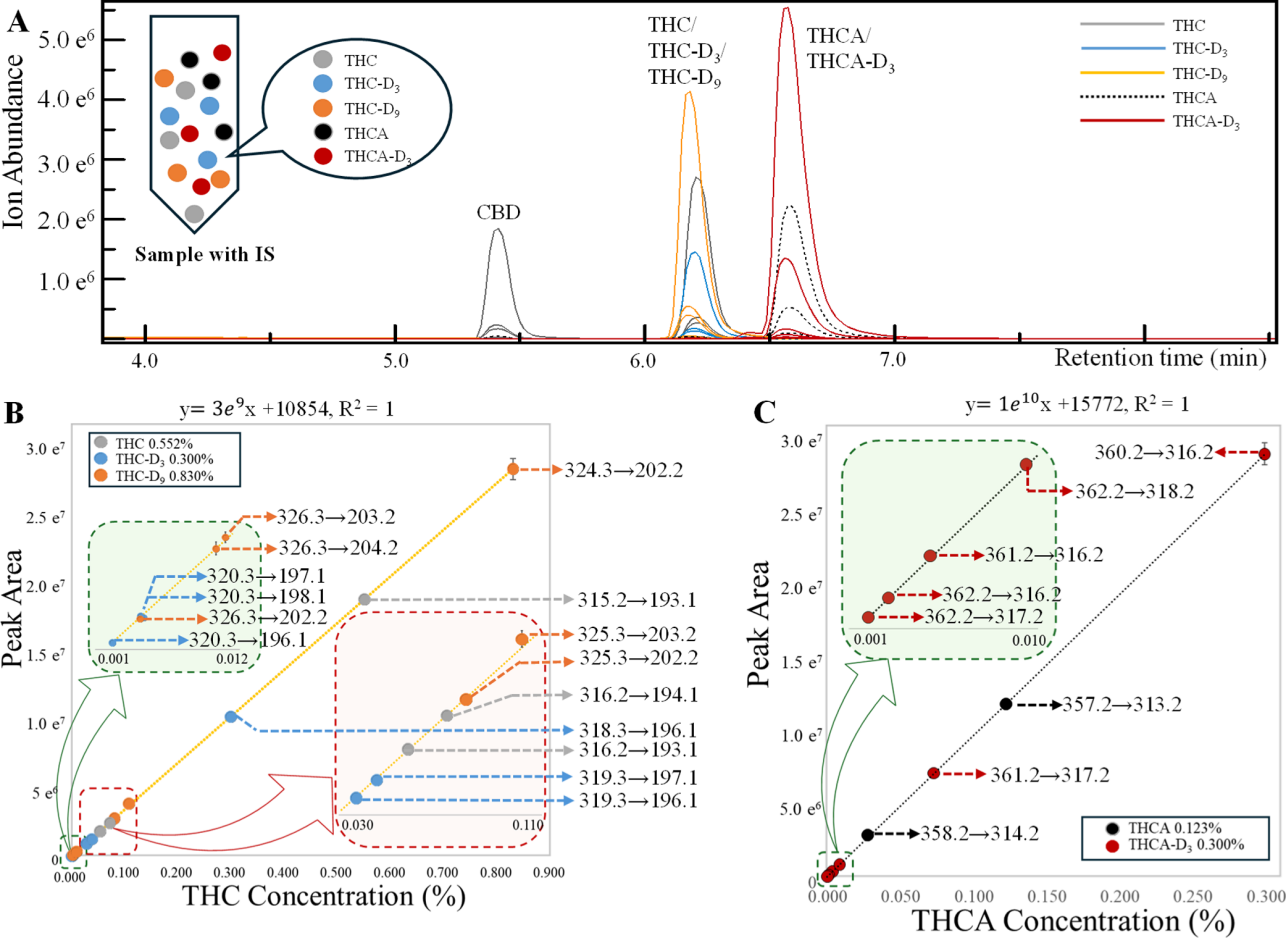
(A) Representative chromatograms of the sample spiked with CBD (0.5%, 2.5 μg/mL), THC (0.552%, 2.76 μg/mL), THC-D_3_ (0.3%, 1.5 μg/mL), THC-D_9_ (0.830%, 4.15 μg/mL), THCA (0.123%, 0.615 μg/mL), and THCA-D_3_ (0.3%, 1.5 μg/mL). (B) ISCC for THC using the combined MIRM transitions of THC-D_3_ and THC-D_9_. Gray markers show concentrations calculated from native-isotopologue THC transitions. (C) ISCC for THCA using the MIRM transitions of THCA-D_3_. Black markers show concentrations calculated from native-isotopologue THCA transitions.

Figure 2A shows the chromatographic separation of native analytes (THC, THCA, CBD) together with their stable-isotope-labeled internal calibrators (THC-D_3_, THC-D_9_, THCA-D_3_), all of which were mixed directly into the same sample prior to LC–QQQ–MS analysis. In this workflow, the stable-isotope-labeled analogs (THC-D_3_, THC-D_9_, THCA-D_3_) serve as internal calibrators added at known concentrations, whereas the native analytes (THC and THCA) act as the unknown target analytes to be quantified. Under the chromatographic conditions used, all compounds eluted as baseline-resolved peaks, ensuring that no isomeric or chromatographic overlap occurred among labeled and unlabeled species. The stable isotope-labeled analogs coeluted with their unlabeled counterparts, and no isotope-dependent retention shift was observed within the method’s chromatographic resolution. For each compound, the specific MIRM isotopologue transitions listed in Table S3 were monitored.

For THC, two SIL analogs (THC-D_3_ and THC-D_9_) were intentionally included at different spiking levels to extend the useable calibration range. This design is essential because THC concentrations in real-world samples can vary by several orders of magnitude across matrices; using two SIL levels ensures that at least one isotopologue set produces signals within the optimal dynamic range of the ISCC. In addition, the isotopologue transitions of native THC and THCA were also recorded. At high native-analyte concentrations, the primary transition of unlabeled THC or THCA may still exceed the linear dynamic range. In such cases, their lower-abundance native isotopologue transitions remain within range and can be used as alternative quantitative channels.

Because the ISCC approach uses labeled calibrators to quantify native analytes, the method assumes that labeled and unlabeled counterparts exhibit identical MS responses at equal molar concentrations. In practice, however, small but systematic differences may arise due to isotopic substitution, instrument tuning, or differences in the purity of standard materials. To account for these effects, a correction factor (α) is applied. The factor is determined empirically by comparing the response of the labeled calibrator with that of the unlabeled analyte at the same molar concentration (detailed in Supporting Information: *Calculation of Correction Factor*). Because THC and THCA concentrations are ultimately reported in weight-percent units, the comparison between labeled and unlabeled species must initially be performed on a molar basis, and the resulting correction factor is then used to convert ISCC-derived molar equivalents into weight-based concentrations.

After applying the correction factor to harmonize the responses of labeled and unlabeled analytes, the MIRM transitions from THC-D_3_ and THC-D_9_ converge onto a single calibration line, yielding an effective dynamic range of ∼690-fold for THC (Figure 2B). In parallel, concentrations of native THC were also computed from its own isotopologue transitions, shown as gray markers in Figure 2B, fell precisely along the same regression line established by the two SIL calibrators. This agreement demonstrates that the native THC isotopologue channels provide valid quantitative points even when the monoisotopic transition is saturated. Thus, when THC concentrations in unknown samples exceed the upper limit of the calibration by using its monoisotopic transition, lower-abundance THC isotopologue channels can still be used for accurate quantitation without additional dilution, effectively extending the useable dynamic range of the method. A similar outcome was observed for THCA, where the THCA-D_3_-derived transitions yielded an ISCC spanning ∼370-fold, and the isotopologue-derived concentrations of native THCA aligned closely with this calibration (Figure 2C).

Here, the reported ∼690-fold (THC) and ∼370-fold (THCA) dynamic ranges refer to the SIL-derived ISCC regression range (THC-D_3_/THC-D_9_ and THCA-D_3_, respectively). At higher native-analyte abundances, lower-intensity native isotopologue transitions can provide additional quantification points beyond monoisotopic saturation, thereby extending the practical range without requiring further dilution. An additional advantage of monitoring multiple isotopologue transitions is increased resilience to unexpected matrix interferences. If a particular MIRM transition becomes compromised by coeluting species in an unknown sample, alternative isotopologue channels, whether from the SIL calibrators or the native analytes, can be substituted without loss of quantitative accuracy. This built-in redundancy enhances the robustness and versatility of the ISCC–MIRM workflow, ensuring reliable quantitation even in highly heterogeneous or unpredictable matrices. Together, these findings demonstrate that both labeled and unlabeled isotopologue transitions produce consistent quantitative responses, enabling a robust internally generated calibration curve from a single injection without reliance on external standards.

### Matrix Effects Across Cannabis-Derived Products

Matrix effects were evaluated across six representative sample types, including plant, gummy, oil, wax, cream, and a dietary supplement, by comparing the responses of the stable-isotope-labeled internal calibrators (THC-D_3_, THC-D_9_, THCA-D_3_) in each matrix relative to a solvent control. Some sample extracts (e.g., plants) showed low-level endogenous THC and THCA, so only the SIL calibrator transitions were used for matrix-effect calculations. To isolate ion suppression or enhancement independently of extraction efficiency, the SIL calibrators were added after extraction, and the resulting responses were expressed as relative signal intensities with the solvent set to 100%. As shown in Figure 3A, all matrices produced varying degrees of ion suppression, with gummies exhibiting the strongest effect (58%, 59%, and 38% of the solvent signal for THC-D_3_, THC-D_9_, and THCA-D_3_, respectively). The remaining matrices (oil, wax, cream, dietary supplement) showed moderate suppression patterns consistent with their chemical complexity. Despite these matrix-dependent intensity differences, the ISCCs constructed from the SIL calibrators maintained excellent linearity in every matrix (*R* ^2^ > 0.999; Figure 3B,C). Although the slopes differed across matrices, reflecting varying degrees of ion suppression, the linear correlation between the theoretical concentration equivalents (derived from the SIL isotopologue transitions) and their measured peak areas was preserved. Because isotopic abundances scale proportionally under uniform suppression, the slope changes do not impair quantitation. This behavior demonstrates that the ISCC approach inherently compensates for matrix effects because the calibration curve is generated inside each sample. As a result, differences in ion suppression do not translate into quantification bias, enabling reliable measurement of native THC and THCA across chemically diverse sample types without the need for matrix-matched external standards.

**Figure 3:**
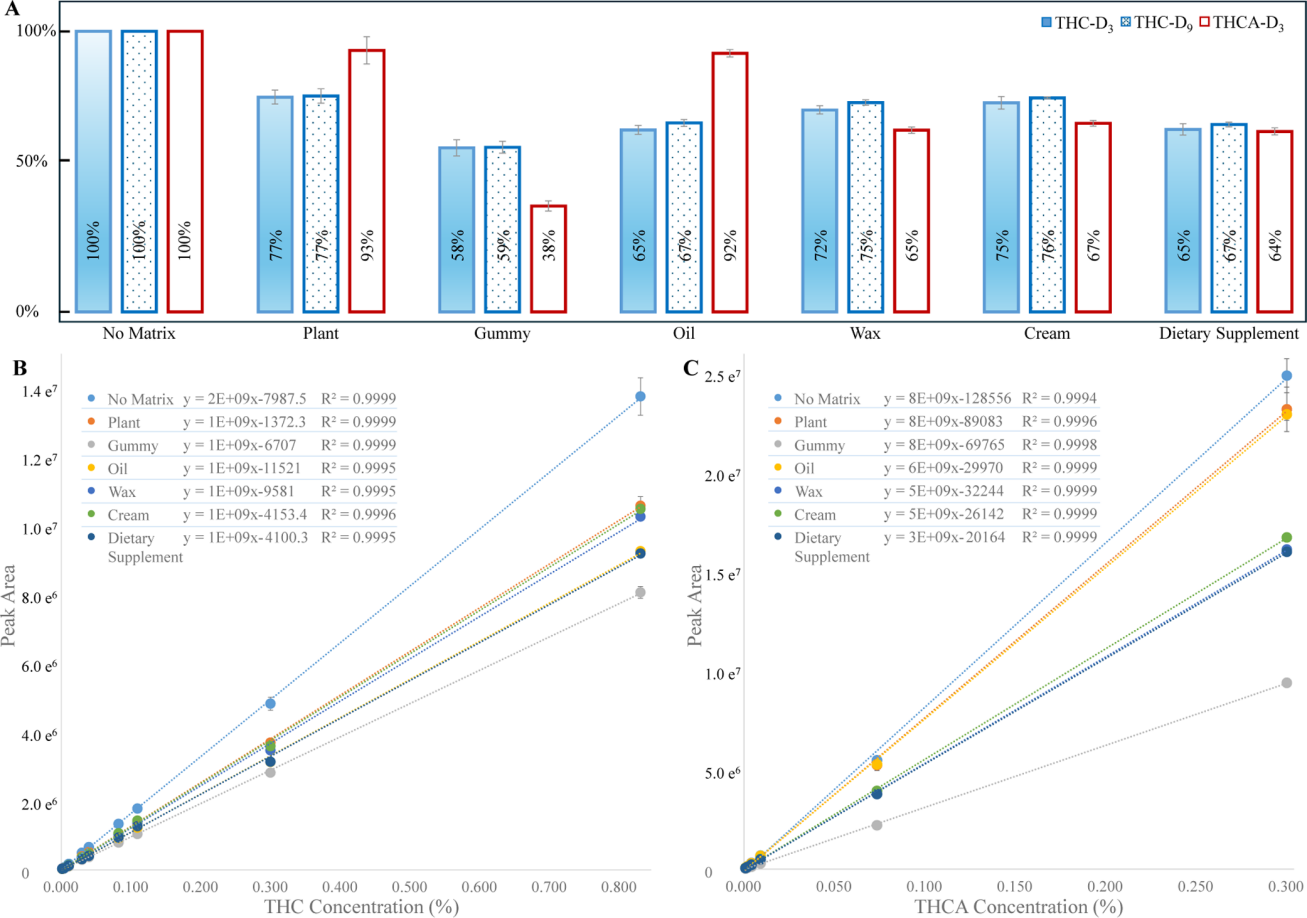
(A) Relative peak area ratios of the most abundant MIRM transitions of THC-D_3_, THC-D_9_, and THCA-D_3_ measured in solvent and six sample matrices. The solvent response is normalized to 100%. (B) ISCCs for THC constructed from THC-D_3_ and THC-D_9_ transitions in solvent and each matrix. (C) ISCCs for THCA constructed from THCA-D_3_ transitions in solvent and each matrix (*n* = 3). Note: details of MIRM transitions for THC-D_3_, THC-D_9_, and THCA-D_3_ are labeled in Figure 2.

### Precision and Accuracy of ISCC Quantification

The precision and accuracy of the ISCC method were evaluated in two representative matrices including CBD oil and CBD gummy, using three spike levels of THC and THCA (0.030%, 0.10%, and 0.30%). As summarized in Table 1, concentrations calculated from the monoisotopic MIRM transitions closely matched the spiked values across all levels, with precision (% RSD) consistently below 10%. Accuracy, expressed as percent deviation from the true concentration, remained within ±10% for both analytes in both matrices. These results demonstrate that the ISCC approach provides reliable quantitative performance over a broad concentration range and across chemically distinct matrices. Importantly, the method maintains accuracy and precision without requiring external calibration curves or matrix-matched standards, supporting its suitability for routine quantification of THC and THCA in diverse cannabis-derived products.

**Table 1:** Precision (% RSD) and Accuracy (% Deviation from True Value) of Spiked THC and THCA in the CBD Oil and Gummy Samples Using Monoisotopic MIRM Transitions^a^ (*n* = 3)

	spiked THC and THCA	calculated THC (%)	precision (%)	accuracy (%)	calculated THCA (%)	precision (%)	accuracy (%)
oil	low (0.030%)	0.029	3.8	–4.9	0.032	4.8	7.6
	medium (0.10%)	0.094	7.3	–6.0	0.108	1.3	8.3
	high (0.30%)	0.310	2.0	3.2	0.312	1.5	4.0
gummy	low (0.030%)	0.028	3.0	–6.1	0.030	3.5	1.4
	medium (0.10%)	0.110	2.1	9.7	0.110	1.7	9.9
	high (0.30%)	0.294	2.4	–2.0	0.299	1.8	0.0

a 315.2 > 193.1 for THC quantitation and 357.2 > 313.2 for THCA quantitation.

Accuracy and precision were also assessed using the secondary MIRM transitions of THC and THCA (Table S5). These transitions yielded satisfactory performance, with accuracy within ±15% and precision ≤15% for both analytes. This finding highlights the utility of native isotopologue channels for accurate quantitation when concentrations exceed the upper limit of the primary transition or when the monoisotopic transition is affected by unexpected matrix interferences.

### Quantitative Application of ISCC to Commercial Matrices

The applicability of the ISCC strategy was evaluated using a range of commercial CBD products, including oil, wax, cream, gummy, and a dietary supplement, as well as two cannabis-derived plant materials. Samples were fortified across a broad concentration range using the levels listed in Table S2. Concentrations of THC and THCA obtained from the ISCC method were compared with those determined by a conventional external calibration approach (Figure 4). Across all matrices, the ISCC-derived concentrations closely aligned with the 1:1 identity line, demonstrating excellent agreement between ISCC and external calibration in both the low (0.005–0.025%) and higher (0.025–0.625%) concentration ranges. This concordance was maintained even in matrices known to cause substantial ion suppression, such as gummies, creams, and waxes, indicating that the ISCC approach successfully compensates for matrix-dependent variability by constructing a calibration curve directly within each sample. This is particularly relevant for forensic compliance laboratories in which sample heterogeneity and throughput demands prohibit routine preparation of matrix-matched calibrations.

**Figure 4:**
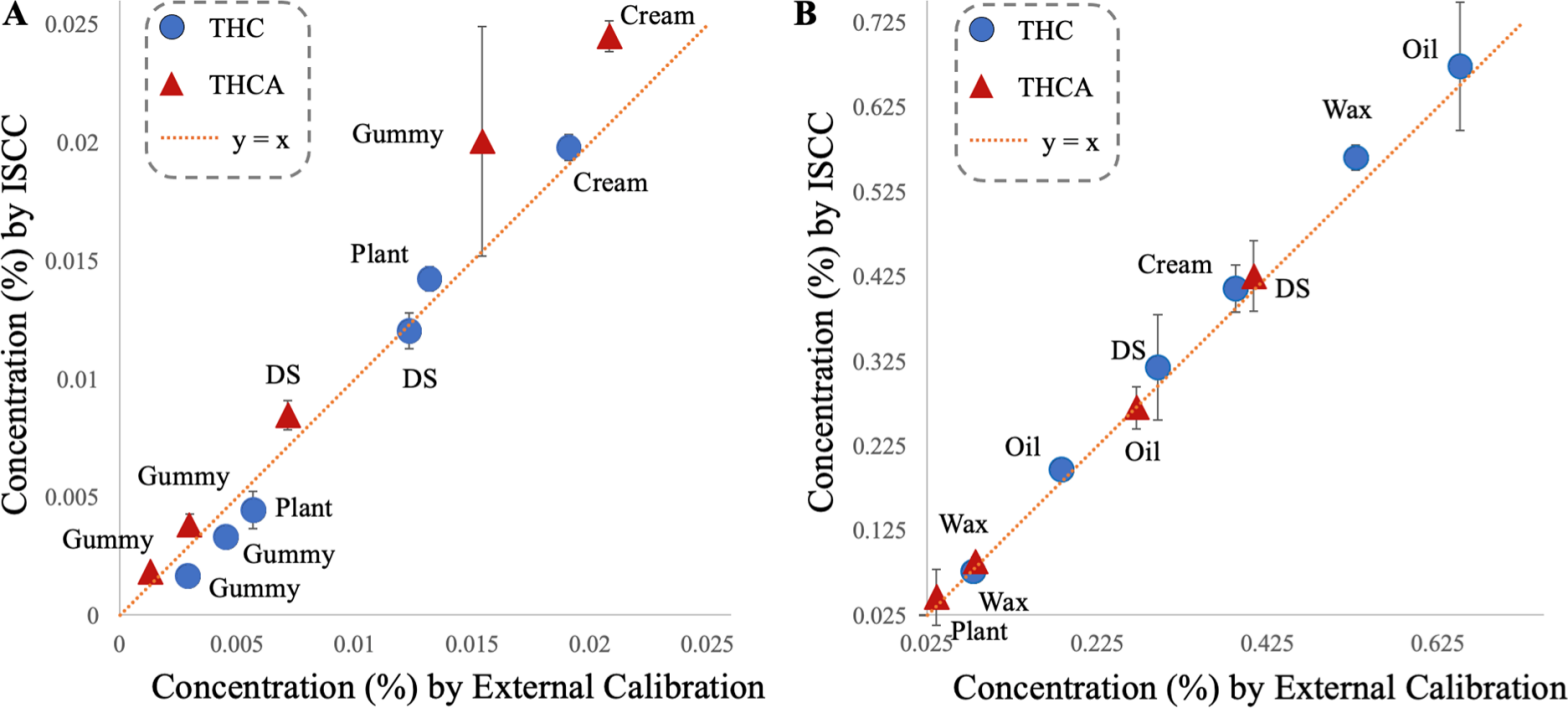
Quantitation of THC and THCA in various sample matrices using the ISCC method versus external calibration. (A) Low-level range: 0.005% to 0.025%. (B) High-level range: 0.025% to 0.625% (*n* = 3).

Beyond its quantitative accuracy, the ISCC method offers several practical advantages for the routine analysis of cannabis-derived products. The approach eliminates the need for external calibration standards and matrix-matched curves, reduces the number of injections required, and minimizes the labor associated with preparing and validating multiple standard solutions. Because the calibration is internally generated from the isotopologue transitions of the SIL calibrators, the method remains robust across diverse product types and concentration ranges without additional optimization. The strong agreement between ISCC and external calibration in commercial samples confirms that the ISCC workflow provides a reliable, matrix-tolerant, and operationally efficient strategy for quantifying THC and THCA in real-world forensic and regulatory applications. This makes ISCC particularly attractive for forensic cannabis testing, where diverse and evolving product matrices, variable THC/THCA content, and the need for regulatory compliance create substantial analytical challenges.

Although this proof-of-concept used one MRM → MIRM transition series for each analyte, THC and THCA generate additional product ions under CID (i.e., 315.2 > 123.1 for THC^17^ and 359.2 > 219.1 for THCA^18^). Each provides its own isotopologue family, offering the potential to integrate multitransition ISCCs. Future work will evaluate combining these orthogonal MIRM sets to further extend linear dynamic range, increase tolerance to transition-specific interferences, and improve overall methodological robustness. Secondary MIRM transitions produced comparable quantitation performance (Table S5), suggesting minimal transition-specific bias under the present conditions. For future multitransition ISCC implementations, correction factors may need to be evaluated per transition family because isotope effects and fragmentation efficiencies can vary across product-ion channels.

## Conclusions

In this study, we developed an in-sample calibration curve (ISCC) strategy based on multiple isotopologue reaction monitoring for the quantification of THC and THCA in cannabis-derived products. By leveraging the theoretical relative isotopic abundances of stable-isotope-labeled calibrators, the ISCC method generates multiple internal calibration points within each injection, eliminating the need for external calibration curves and matrix-matched standards. The incorporation of two SIL calibrators for THC (THC-D_3_ and THC-D_9_), together with a correction-factor procedure to harmonize labeled-unlabeled response differences, enabled quantification across a >600-fold dynamic range, addressing the extreme concentration variability encountered in real-world samples. The ISCC approach demonstrated excellent linearity, precision, and accuracy across diverse matrices, including plant material, edibles, oils, waxes, creams, and dietary supplements. Matrix-effect evaluation showed that the internally generated calibration curve effectively compensates for ion suppression and signal variability, producing consistent results even in highly complex product matrices. Comparison with a conventional external calibration method confirmed strong agreement across all concentration levels and sample types, underscoring the robustness and reliability of the ISCC workflow. ISCC is a calibration strategy rather than a separation mechanism; therefore, differentiation of Δ^9^-THC from other positional isomers, such as Δ^8^-THC and Δ^10^-THC, depends on chromatographic resolution. The ISCC workflow can be extended to Δ^8^-THC and Δ^10^-THC quantification, provided that adequate LC separation (i.e., the HPLC method from the National Institute of Standards and Technology^19^) and appropriate isotope-labeled standards are employed.

Overall, this work extends the ISCC–MIRM concept into the forensic and consumer product domains and establishes a practical, efficient, and matrix-tolerant calibration strategy for routine cannabinoid analysis. The method substantially reduces analytical burden, improves tolerance to matrix heterogeneity, and offers an attractive alternative to traditional calibration workflows for laboratories responsible for regulatory and forensic testing of cannabis and hemp products.

## Supplementary Material



## Notes

Tables S1–S5; Detailed spiking information for THC, THCA, THC-D_3_, THC-D_9_, and THCA-D_3_ in various samples;MIRM parameters; MIRM transitions and theoretical isotopologue ratios; precision/accuracy using secondary MIRM transitions; correction-factor calculation (PDF)
