# Supplementary material for: Matrix-Tolerant Quantification of THC and THCA in Complex Cannabis Products Using In-Sample Calibration with Multiple Isotopologue Reaction Monitoring
Source: J Am Soc Mass Spectrom. 2026 Jan 26;37(2):548–55. doi: 10.1021/jasms.5c00439 (PMC12879920; doi:10.1021/jasms.5c00439)
Supplement: Supplementary file 1 [file js5c00439_si_001.pdf]

Supplemental Material

Matrix-Tolerant Quantification of THC and THCA in Complex Cannabis Products Using In-Sample Calibration with Multiple Isotopologue Reaction Monitoring

Yanfang Li, Mengliang Zhang*

Department of Chemistry and Biochemistry, Ohio University, Athens, OH, 45701

*Corresponding authors: Mengliang Zhang, *E-mail address:* zhangm4@ohio.edu. Phone: +01 740-593-0719

Calculation of Correction Factor

To account for small response differences between labeled and unlabeled analytes arising from deuterium substitution, a correction factor (α) was applied prior to ISCC construction. The correction factor was calculated by comparing the response of the native analyte with that of its corresponding SIL calibrator at equivalent theoretical concentration. For THC, α was obtained using the relationship:

$$\frac{C_{\text{THC}}}{A_{\text{THC}}} = \frac{C_{\text{THC-D3}}}{A_{\text{THC-D3}}} \times \alpha,$$

where C_{THC} and $C_{\text{THC-D3}}$ represent the theoretical concentration equivalents derived from isotopologue abundances, and A_{THC} and $A_{\text{THC-D3}}$ represent the corresponding MIRM peak areas. A second correction factor (α′) was similarly calculated using THC-D_9:

$$\frac{C_{\text{THC-D9}}}{A_{\text{THC-D9}}} = \frac{C_{\text{THC-D3}}}{A_{\text{THC-D3}}} \times \alpha'.$$

For illustration, the working solution contained 0.300% THC-D_3, 1.00% THC-D_9, and 0.500% THC. After applying α and α′, the corrected concentration equivalents for THC-D_9 and THC were 0.830% and 0.552%, respectively. These corrected values were then used for ISCC construction to ensure that labeled and unlabeled analytes were quantitatively comparable, independent of isotopic substitution or purity differences. It is noted that α typically ranges close to unity.

Table S1. CBD oil and gummy samples spiked with different amounts of THC, THCA, THC-D_3, THC-D_9, and THCA-D_3.

Sample Name	Sample Weight/mg	Volume/µL				
		THC (1 mg/mL)	THCA (1 mg/mL)	THC-D_3 (100 µg/mL)	THC-D_9 (1 mg/mL)	THCA-D_3 (100 µg/mL)
oil spike 0.3%THC and THCA-1	10.8	30	30	300	100	300
oil spike 0.3%THC and THCA-2	10.6	10	10	300	100	300
oil spike 0.3%THC and THCA-3	10.5	3	3	300	100	300
gummy spike 0.3%THC and THCA-1	10.2	30	30	300	100	300
gummy spike 0.3%THC and THCA-2	10.8	10	10	300	100	300
gummy spike 0.3%THC and THCA-3	10.3	3	3	300	100	300

Table S2. Commercial CBD products and cannabis-derived plant samples spiked with different amounts of THC and THCA.

Sample Name	Sample Weight/mg	Volume/μL	
		THC (1 mg/mL)	THCA (1 mg/mL)
oil spike 1%THC-1	10.7	100	0
oil spike 1%THC-2	10.5	100	0
oil spike 1%THC-3	10.8	100	0
wax spike 0.8%THC-1	10.3	80	0
wax spike 0.8%THC-2	11.0	80	0
wax spike 0.8%THC-3	11.2	80	0
cream spike 0.6%THC-1	10.1	60	0
cream spike 0.6%THC-2	10.1	60	0
cream spike 0.6%THC-3	10.2	60	0
DS spike 0.5%THC and THCA-1	10.6	50	50
DS spike 0.5%THC and THCA-2	10.2	50	50
DS spike 0.5%THC and THCA-3	10.8	50	50
oil spike 0.3%THC and THCA-1	10.1	30	30
oil spike 0.3%THC and THCA-2	10.5	30	30
oil spike 0.3%THC and THCA-3	10.7	30	30
wax spike 0.1%THC and THCA-1	10.5	10	10
wax spike 0.1%THC and THCA-2	10.8	10	10
wax spike 0.1%THC and THCA-3	10.8	10	10
cream spike 0.03%THC and THCA-1	10.7	3	3
cream spike 0.03%THC and THCA-2	10.7	3	3
cream spike 0.03%THC and THCA-3	10.9	3	3
DS spike 0.01%THC and THCA-1	10.4	1	1
DS spike 0.01%THC and THCA-2	10.7	1	1
DS spike 0.01%THC and THCA-3	11.3	1	1
gummy spike 0.005%THC and THCA-1	11.4	0.5	0.5
gummy spike 0.005%THC and THCA-2	10.6	0.5	0.5
gummy spike 0.005%THC and THCA-3	11.6	0.5	0.5
gummy spike 0.003%THC and THCA-1	10.6	0.3	0.3
gummy spike 0.003%THC and THCA-2	11.0	0.3	0.3
gummy spike 0.003%THC and THCA-3	10.6	0.3	0.3
Plant_A-1	10.1	0	0
Plant_A-2	10.8	0	0
Plant_A-3	10.8	0	0
Plant_B-1	10.8	0	0
Plant_B-2	10.6	0	0
Plant_B-3	10.9	0	0

“DS” denotes dietary supplements.

Table S3. MIRM parameters.

Transitions	Precursor (*m/z*)	Product (*m/z*)	Dwell time (msec)	Q1 (V)	CE	Q3 (V)
THC ($C_{21}H_{30}O_2$)						
315.2>193.1	315.2	193.1	30.0	-16.0	-23.0	-20.0
316.2>193.1	316.2	193.1	30.0	-16.0	-23.0	-20.0
316.2>194.1	316.2	194.1	30.0	-16.0	-23.0	-20.0
317.2>193.1	317.2	193.1	30.0	-16.0	-23.0	-20.0
317.2>194.1	317.2	194.1	30.0	-16.0	-23.0	-20.0
317.2>195.1	317.2	195.1	30.0	-16.0	-23.0	-20.0
THC-D3 ($C_{21}H_{27}D_3O_2$)						
318.3>196.1	318.3	196.1	30.0	-16.0	-23.0	-20.0
319.3>196.1	319.3	196.1	30.0	-16.0	-23.0	-20.0
319.3>197.1	319.3	197.1	30.0	-16.0	-23.0	-20.0
320.3>196.1	320.3	196.1	30.0	-16.0	-23.0	-20.0
320.3>197.1	320.3	197.1	30.0	-16.0	-23.0	-20.0
320.3>198.1	320.3	198.1	30.0	-16.0	-23.0	-20.0
THC-D9 ($C_{21}H_{21}D_9O_2$)						
324.3>202.2	324.3	202.2	30.0	-16.0	-23.0	-20.0
325.3>202.2	325.3	202.2	30.0	-16.0	-23.0	-20.0
325.3>203.2	325.3	203.2	30.0	-16.0	-23.0	-20.0
326.3>202.2	326.3	202.2	30.0	-16.0	-23.0	-20.0
326.3>203.2	326.3	203.2	30.0	-16.0	-23.0	-20.0
326.3>204.2	326.3	204.2	30.0	-16.0	-23.0	-20.0
THCA ($C_{22}H_{30}O_4$)						
357.2>313.2	357.2	313.2	30.0	19.0	24.0	20.0
358.2>313.2	358.2	313.5	30.0	19.0	24.0	20.0
358.2>314.2	358.2	314.2	30.0	19.0	24.0	20.0
359.2>313.2	359.2	313.2	30.0	19.0	24.0	20.0
359.2>314.2	359.2	314.2	30.0	19.0	24.0	20.0
359.2>315.2	359.2	315.2	30.0	19.0	24.0	20.0
THCA-D3 ($C_{22}H_{27}D_3O_4$)						
360.2>316.2	360.2	316.2	30.0	19.0	24.0	20.0
361.2>316.2	361.2	316.2	30.0	19.0	24.0	20.0
361.2>317.2	361.2	317.2	30.0	19.0	24.0	20.0
362.2>316.2	362.2	316.2	30.0	19.0	24.0	20.0
362.2>317.2	362.2	317.2	30.0	19.0	24.0	20.0
362.2>318.2	362.2	318.2	30.0	19.0	24.0	20.0

Table S4. MIRM transitions and their theoretical ratio of THC-D_3, THC-D_9, THCA-D_3, THC, and THCA.

Transitions	Precursor (*m/z*)	Product (*m/z*)	Neutral loss (Da)	Isotopic distribution (%)*		Theoretical isotopic abundances (%)#	Theoretical concentration equivalent$
				Neutral loss (△)	Product (□)		
THC-D_3 ($C_{21}H_{27}D_3O_2$, 0.300%**)							
**318.3>196.1**	318.3	196.1	122.1	100.00	100.00	100.00	0.300%
319.3>196.1	319.3	196.1	123.1	9.90	100.00	9.90	0.030%
319.3>197.1	319.3	197.1	122.1	100.00	13.22	13.22	0.040%
320.3>196.1	320.3	196.1	124.1	0.44	100.00	0.44	0.001%
320.3>197.1	320.3	197.1	123.1	9.90	13.22	1.31	0.004%
320.3>198.1	320.3	198.1	122.1	100.00	1.21	1.21	0.004%
THC-D_9 ($C_{21}H_{21}D_9O_2$, 1.000%**)							
**324.3>202.2**	324.3	202.2	122.1	100.00	100.00	100.00	1.000%
325.3>202.2	325.3	202.2	123.1	9.90	100.00	9.90	0.099%
325.3>203.2	325.3	203.2	122.1	100.00	13.15	13.15	0.131%
326.3>202.2	326.3	202.2	124.1	0.44	100.00	0.44	0.004%
326.3>203.2	326.3	203.2	123.1	9.90	13.15	1.30	0.013%
326.3>204.2	326.3	204.2	122.1	100.00	1.21	1.21	0.012%
THCA-D_3 ($C_{22}H_{27}D_3O_4$, 0.300%**)							
**360.2>316.2**	360.2	316.2	44.0	100.00	100.00	100.00	0.300%
361.2>316.2	361.2	316.2	45.0	1.16	100.00	1.16	0.004%
361.2>317.2	361.2	317.2	44.0	100.00	23.09	23.09	0.073%
362.2>316.2	362.2	316.2	46.0	0.41	100.00	0.41	0.002%
362.2>317.2	362.2	317.2	45.0	1.16	23.09	0.27	0.001%
362.2>318.2	362.2	318.2	44.0	100.00	2.95	2.95	0.009%
THC ($C_{21}H_{30}O_2$, 0.500%**)							
**315.2>193.1**	315.2	193.1	122.1	100.00	100.00	100.00	0.500%
316.2>193.1	316.2	193.1	123.1	9.90	100.00	9.90	0.049%
316.2>194.1	316.2	194.1	122.1	100.00	13.25	13.25	0.066%

317.2>193.1	317.2	193.1	124.1	0.44	100.00	0.44	0.002%
317.2>194.1	317.2	194.1	123.1	9.90	13.25	1.31	0.007%
317.2>195.1	317.2	195.1	122.1	100.00	1.22	1.22	0.006%
THCA ($C_{22}H_{30}O_4$, 0.100%**)							
**357.2>313.2**	357.2	313.2	44.0	100.00	100.00	100.00	0.100%
358.2>313.2	358.2	313.5	45.0	1.16	100.00	1.16	0.001%
358.2>314.2	358.2	314.2	44.0	100.00	23.12	23.12	0.023%
359.2>313.2	359.2	313.2	46.0	0.41	100.00	0.41	0.000%
359.2>314.2	359.2	314.2	45.0	1.16	23.12	0.27	0.000%
359.2>315.2	359.2	315.2	44.0	100.00	2.96	2.96	0.003%

* Isotopic distribution was obtained through online calculator; ** spiked concentration; # Theoretical isotopic abundances = % ∆ × % of □; $ Theoretical concentration equivalent = Theoretical isotopic abundances × spiked concentration. The primary monoisotopic transitions are bold.

Table S5. Precision (% RSD) and Accuracy (% Deviation from True Value) of Spiked THC and THCA in the CBD Oil and Gummy Samples Using Secondary MIRM Transitions* (n = 3)

	Spiked THC and THCA	Calculated THC	Precision	Accuracy	Calculated THCA	Precision	Accuracy
Oil	Low (0.030%)	0.026%	9.8%	-14.1%	0.032%	13.6%	7.2%
	Medium (0.10%)	0.113%	1.6%	13.2%	0.113%	1.2%	12.7%
	High (0.30%)	0.276%	2.7%	-7.9%	0.320%	1.7%	6.8%
Gummy	Low (0.030%)	0.029%	1.2%	-4.7%	0.032%	2.4%	6.0%
	Medium (0.10%)	0.102%	1.3%	2.0%	0.115%	0.8%	15.0%
	High (0.30%)	0.291%	2.2%	-2.8%	0.313%	1.6%	4.4%

* 316.2>193.1 for THC quantitation and 358.2>314.2 for THCA quantitation.
